# Multi-barrier field-emission behavior in PBTTT thin films at low temperatures

**DOI:** 10.1038/srep08396

**Published:** 2015-02-11

**Authors:** Evan S. H. Kang, Eunseong Kim

**Affiliations:** 1 Center for Supersolid and Quantum matter Research, Korea Advanced Institute of Science and Technology, Deajeon 305–701 (Korea)

## Abstract

We investigated the low-temperature transport mechanism for poly[2,5-bis(3-alkylthiophen-2-yl)thieno(3,2-b)thiophene] (PBTTT). The temperature-dependent transport behavior was studied by varying the drain–source electric field and gate bias. The results suggest that low-temperature charge transport is dominated by direct tunneling at low electric fields, while field emission is prevailing for high electric fields with high carrier densities. However, the obtained barrier heights are remarkably greater than expected in a conventional field emission. We propose a simplified model of field emission through quasi-one-dimensional path with multiple barriers which shows good agreement with the results more clearly. Field emission across the domain boundaries may assist in overcoming the transport barriers induced by the interchain disorder, which results in the weak temperature dependence of conductivities and nonlinear current–voltage relation at low temperatures.

Understanding the charge transport mechanism in semiconducting polymers is essential to advance the applications of organic electronics^1^,^2^,^3^,^4^. Low-temperature experiments have revealed the transport mechanism in semiconducting polymer systems^3^,^4^,^5^,^6^,^7^,^8^. Recently, Yuen et al. reported that the low-temperature transport in poly[2,5-bis(3-alkylthiophen-2-yl)thieno(3,2-b)thiophene] (PBTTT) at high carrier densities can be described by the one-dimensional (1d) Luttinger liquid (LL) theory, rather than in the framework of the classical transport theory^3^. The current–voltage (I–V) characteristics obtained in a wide range of parameters (e.g. temperature, gate-induced carrier density, and drain–source voltage) show good agreement with universal scaling derived using the LL theory. Because of the complex microstructure of the semicrystalline polymer thin films, the charge transport inevitably involves the penetration through a number of polymers. Accordingly, the overall transport behavior should include both interchain and intrachain transports. For true 1d transport, however, the interchain transport contribution should be negligible such that the intrachain transport along the 1d path composed of polymer chains in series becomes dominant. The origin of the dramatic 1d transport was not clearly elucidated in Yuen's work^3^. Instead, this 1d transport phenomena were attributed to the extraordinary microstructural nature of the PBTTT thin films.

More recently, Worne et al. determined that similar universal scaling can be established in the transport measurements of poly(3-hexylthiophene) (P3HT) and 6,13-bis(triisopropyl-silylethynyl) (TIPS) pentacene. Scaling behavior is not expected, because these organic thin films do not exhibit significant microstructural orders as PBTTT does^9^. TIPS-pentacene, in particular, is a small molecule without any chain structure that forms molecular crystals bonded through van der Waals forces. In such cases, a 1d LL transport path is not feasible. Essentially identical discussions were also presented for poly(3,4-ethylenedioxythiophene)–poly(4-styrenesulfonic acid) (PEDOT:PSS)^10^. PEDOT:PSS thin films consist of three-dimensional spherical amorphous grains, and its highly disordered characteristics do not allow LL transport. Instead, weak Coulomb blockade is believed to be a possible transport mechanism for describing the scaling behavior in PEDOT:PSS. Moreover, it has been shown that the universal scaling can be also mimicked by other transport mechanisms such as variable range hopping (VRH) in quasi-1d systems^11^,^12^ and polaron hopping by nuclear tunneling^13^. Therefore, the universal scaling-like behavior can be explained without invoking 1d LL transport, and the origin of the low-temperature transport behavior in PBTTT remains unclear.

Herein, we investigate the transport mechanism of PBTTT at low temperatures. We found that field emission is the dominant low-temperature transport mechanism at high carrier density and high electric fields, whereas direct tunneling is prominent at low fields. A naïve approach with single-barrier field emission was first adopted to explain the tunneling behavior, but the tunneling probability was found to be extremely small for our device configuration. Assuming that the penetration of the series of quasi-domains becomes a dominant transport mechanism at high fields, we employ a simple model describing the field emission through multiple domain boundaries. Within the framework of multi-barrier field emissions, the average domain size and barrier height can be calculated. Finally, the universal scaling is reconstructed using the I–V characteristics in the high field and low temperature regime where field emission is dominant.

## Results

### Low-temperature transport mechanisms

**Figure 1:**
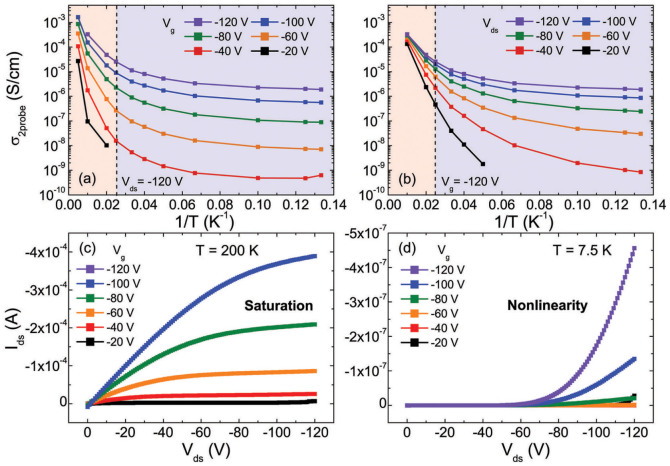
Temperature-dependent conductivities with (a) various *V*_g_ and fixed *V*_ds_ ( = −120 V) and (b) various *V*_ds_ and fixed *V*_g_ ( = −120 V). Output characteristics obtained at (c) 200 K and (d) 7.5 K.

There are two non-classical key features in the low-temperature transport behavior of PBTTT thin films: (1) weak temperature dependence of conductivity and (2) nonlinearity in the I–V characteristics^3^. Both features obtained using PBTTT field effect transistors (FETs) with a 30-μm channel length are shown in Figure 1. Temperature-dependent two-probe conductivities (≡ *I*_ds_/*V*_ds_) with drain–source voltage (*V*_ds_) fixed at −120 V and with gate voltage (*V*_g_) fixed at −120 V are shown in Figure 1a and 1b, respectively. At high temperatures, the conductivities decrease monotonically as the temperature decreases with a relatively large activation energy obtained from the slope in the Arrhenius plot. On the other hand, the temperature dependence becomes weaker with a negligible activation energy for low temperatures below 40 K. This weak temperature dependence indicates that thermal activation is no longer a dominant transport mechanism at low temperatures. Note that the weakly temperature-dependent transport can be achieved with high *V*_ds_ at low temperature ranges, as shown in Figure 1a. On the other hand, clear temperature-dependent transport is still found at low *V*_ds_ even with a high *V*_g_ of −120 V, as shown in Figure 1b. These observations indicate that the strength of *V*_ds_ is more crucial for the appearance of weak temperature dependence than strength of *V*_g_.

Another prominent feature is that the *V*_ds_-dependent conductivity becomes highly nonlinear with decreasing temperature. In the weakly temperature-dependent region in Figure 1b, for instance, increasing *V*_ds_ by a factor of two results in a conductivity increase of orders of magnitudes. This *V*_ds_-dependence of conductivity becomes weaker with increasing temperature. The low-temperature nonlinearity is also revealed in the output characteristics. Typical FET output curves with a saturation region were obtained at 200 K (Figure 1c), while nonlinear I–V curves with an opposite curvature were observed at 7.5 K (Figure 1d).

**Figure 2:**
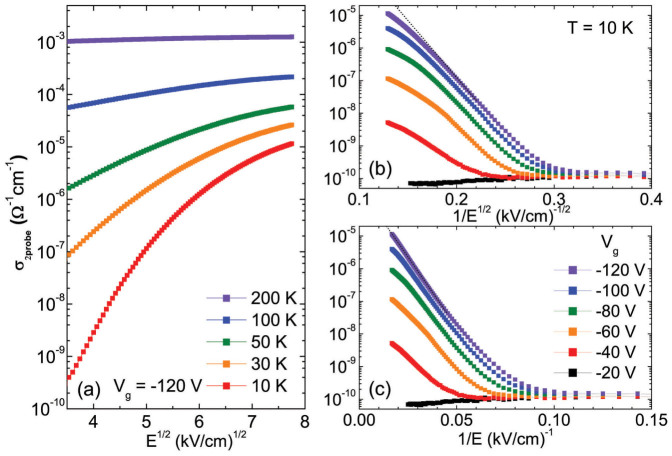
(a) Conductivities as a function of *E*^1/2^ for *V*_g_ = −120 V at various temperatures. Conductivities as a function of (b) *E*^−1/2^ and (c) *E*^−1^ obtained for various *V*_g_ at 10 K.

Both key features indicate that the conductivity is strongly electric-field-dependent in this low-temperature region. To address the appropriate transport mechanism for this nonlinearity, we investigated field-dependent transport models. At relatively high temperatures, the field dependence of conductivity can be described by the Poole–Frenkel relation: 
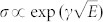
, where *γ* is an empirically obtained coefficient that increases as temperature decreases^9^,^14^. In Figure 2a, the conductivities measured using *V*_g_ of −120 V for various temperatures are delineated as a function of *E*^1/2^. With decreasing temperature, the conductivity becomes nonlinear, thereby demonstrating that the Pool–Frenkel model is not suitable below a certain temperature. The deviation at low temperature leads to the application of other subtle field-dependent models. Previous studies on organic devices have shown that below a critical temperature and above a critical field, the conductivity becomes strongly dependent on the field, i.e., *σ* ∝ exp(−*E*^−1/2^) or *σ* ∝ exp(−*E*^−1^), depending on the transport mechanism in the material^15^. The former relation can be derived from various transport mechanisms such as Efros–Shklovskii VRH^4^,^16^, field-assisted hopping^15^,^17^, or multistep tunneling^9^,^18^,^19^, while the latter relation can be deduced from Fowler–Nordheim field emission^20^,^21^,^22^. To determine the dependence on electric field, the low-temperature conductivities obtained at 10 K for various *V*_g_ are plotted as a function of *E*^−1/2^ and *E*^−1^ in Figure 2b and 2c, respectively. For modest fields, both plots exhibit linear dependences without showing any significant difference. However, for high fields, the conductivity starts to deviate from the linear relation in the *E*^−1/2^ plot, whereas the linear relation is satisfied over the entire range of the electric field in the *E*^−1^ plot. Consequentially, we determined that the dominant transport mechanism in PBTTT thin films at low temperatures for high fields is the field emission.

### Fowler–Nordheim field emission

At sufficiently low temperatures, tunneling becomes the dominant transport mechanism because of the lack of thermal energy for the charge carriers to overcome the barrier. The tunneling probability for charge carriers depends on the shape of the barrier. For a low bias across the barrier, the barrier is rectangular, and the I–V relation is described by the direct tunneling^20^:
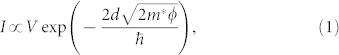
where *ϕ* is the barrier height, *d* is the barrier width, 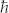 is the reduced Planck's constant, and m* is the effective mass of the charge carrier. When the bias exceeds the barrier height, the barrier becomes triangular, thereby increasing the tunneling probability. This triangular deformation gives rise to Fowler–Nordheim tunneling (i.e. field emission) with the following I–V relation^20^:
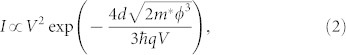
where *q* is the electron charge. The Fowler–Nordheim plot, i.e. ln(*I*/*V*^2^) vs. 1/*V* plot, can distinctly show the transition from direct tunneling to field emission because the equation (1) and (2) can be rearranged in a logarithmic scale, as shown below.
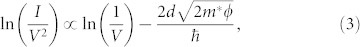

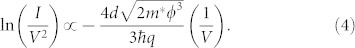
Note that direct tunneling leads to the logarithmic dependence in the Fowler–Nordheim plot, whereas field emission leads to the linear relation with a negative slope. At high temperatures, on the other hand, the dominant transport mechanism is thermally activated hopping, which results in the nearly ohmic output characteristics and subsequently another logarithmic dependence with different coefficients in the Fowler–Nordheim plot^23^.

**Figure 3:**
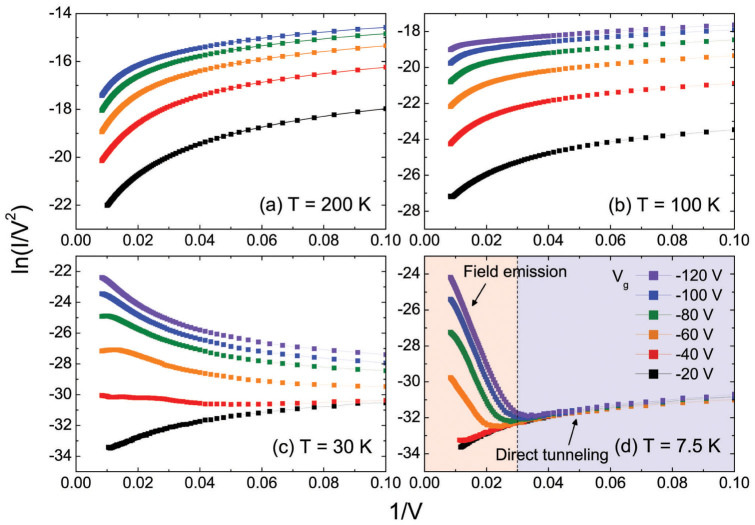
Fowler–Nordheim plots for various temperatures.

Fowler–Nordheim plots for various temperatures summarize the arguments above (Figure 3). At high temperatures, thermally activated hopping is dominant for the entire range of gate biases (Figure 3a and 3b). As temperature decreases, while the logarithmic hopping current is reduced, the additional current with the negative slope (i.e. the evidence of field emission) gradually increases (Figure 3c). Finally, at the lowest temperature (7.5 K), logarithmic curves due to direct tunneling at low bias, straight lines due to field emission at high bias and the transition in between are clearly exhibited in Figure 3d. Note that field emission becomes dominant only in low temperature, high field and high *V*_g_-induced charge density regime. It is worth mentioning that field-emission behavior in organic FETs has also been reported recently^24^, but the crossover from thermally assisted hopping to field emission with decreasing temperature has not been shown yet. Moreover, the transition from tunneling to field emission remarkably resembles that in molecular junction transistors^23^,^25^,^26^.

**Figure 4:**
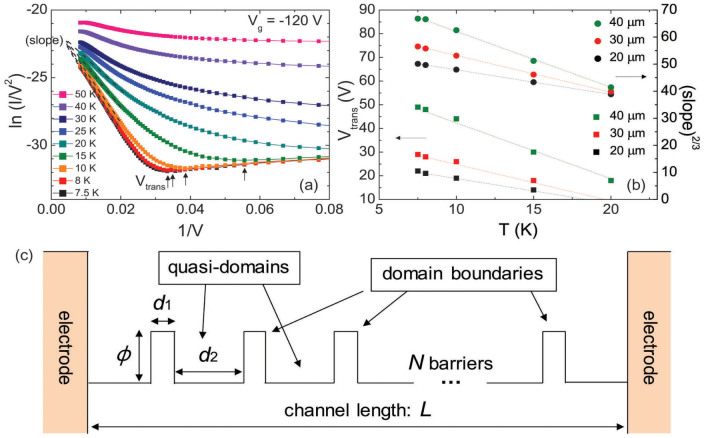
(a) Fowler–Nordheim plots obtained for *V*_g_ = −120 V at low temperatures. *V*_trans_ and the slope are indicated. (b) *V*_trans_ and (slope)^2/3^ as a function of temperature for various channel lengths. (c) Scheme for qausi-1d transport path with multiple barriers.

There are two physically meaningful parameters in the Fowler–Nordheim plots: the transition voltage (*V*_trans_) and slope (
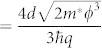
) of the linear relation. *V*_trans_ is defined as the minimum *V*_ds_ at which the field emission (i.e. the negative slope in the Fowler–Nordheim plot) begins. Because *V*_ds_ leads to a Fermi-level difference between the drain and source electrodes and changes the shape of the barrier between them, *V*_trans_ is the minimum voltage at which the shape of the barrier becomes triangular. Therefore, *V*_trans_ is essentially equivalent to the height of the original rectangular barrier^20^. The slope of the linear relation is defined from equation (4), and the (slope)^2/3^ is proportional to the unreduced barrier height (*ϕ*). *V*_trans_ and the slope obtained at a *V*_g_ of −120 V for various temperatures are shown in the Fowler–Nordheim plot (Figure 4a). Both *V*_trans_ and the slope can be clearly defined only at high *V*_ds_ and sufficiently low temperatures without being blurred by thermal activation. *V*_trans_ and the (slope)^2/3^ as a function of temperature for various channel lengths are shown in Figure 4b. The parameters could not be determined in large-gap devices with channel length greater than 50 μm. In fact, we found only direct tunneling with a logarithmic dependence in the Fowler–Nordheim plot without the transition to the field emission, even at the highest *V*_ds_ of −120 V. This is probably due to the small field that developed across the channel. Both parameters exhibit a linear dependence on temperatures with negative slopes, which shows that *V*_trans_ and *ϕ* essentially originate from the same entity, i.e., effective barrier height that is inversely proportional to temperature. Extrapolating the two parameters for *T* = 0 K from the linear fit provides the zero-temperature *V*_trans_ (≡ *V*_trans,0_) and (slope)|*_T_*_→0_ by eliminating thermal effects, and consequently the zero-temperature barrier height can be calculated. However, the calculated values are not consistent with the single-barrier tunneling scheme. First, the barrier height obtained from the *V*_trans_ (tens of volts) is orders of magnitude higher than that obtained from the slope (tens of millivolts). In fact, the *V*_trans_ values in the measurements with PBTTT FETs are orders of magnitude greater than those obtained with molecular junctions (order of few volts^23^,^25^,^26^) or carbon nanotubes (tens of millivolts^27^). Second, we found a smaller *V*_trans_ and (slope)^2/3^ for the devices with a smaller gap (Figure 4b). The strong dependence of *V*_trans_ on the gap size cannot be easily understood, because *V*_trans_ should be only a function of the barrier height and be nearly independent of the barrier width^28^. Moreover, tunneling probability through the channel of tens of micrometers in typical FET configurations would be extremely small^18^.

### Multi-barrier field emission

To resolve the problem related with the unrealistic barrier dimensions, we propose a multi-barrier field-emission model instead of a single-barrier model. For the purpose of simplicity, the multiple barriers are assumed to have the same barrier height, *ϕ*_0_, barrier width, *d*_1_, and inter-distance, *d*_2_. The over-simplified quasi-1d transport path between the source and drain electrodes is schematically depicted in Figure 4c. With these identical equidistant multiple barriers, a single barrier feels the reduced bias, *V*_ds_/*N*, where 
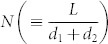
 is the total number of barriers in the channel, *L* is the channel length, and *d*_1_ + *d*_2_ is the quasi-domain size. Then, the transition from tunneling to field emission can be achieved when *V*_ds_/*N* exceeds *ϕ*_0_. Therefore, the relation between *V*_trans,0_ and *ϕ*_0_ can be modified as follows:
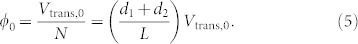


The field-emission I–V relation (equation (4)) should be reconstructed as well. For an individual barrier, *d*_1_, *ϕ*_0_, and *V*_ds_/*N* are substituted for *d*, *ϕ*, and *V*, respectively, which results in a new negative slope in the I–V relation.
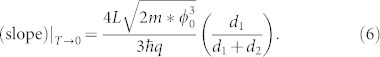


Note that the barrier-dimension discrepancies revealed in the single-barrier assumption can be naturally resolved with this new approach. In the system with multiple barriers, *V*_trans,0_ does not directly indicate a single barrier height but instead the sum of *N* barrier heights. Therefore, *V*_trans,0_ can be much larger than *ϕ*_0_. The channel length dependence of *V*_trans,0_ can be well described with equation (5) as well. The multi-barrier model intrinsically leads to smaller 
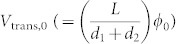
 for smaller channel length *L*. It is also worth noting that because the barrier width is now defined as the width of each barrier, which would be much smaller compared to the channel length, the tunneling probability becomes significant.

Although it is an unsolvable problem with three unknown variables (*d*_1_, *d*_2_, *ϕ*_0_) and two equations (equation (5) and (6)), we can estimate one variable based on the practical measurements and check whether the other values are reasonable or not. Dark-field transmission electron microscopy (TEM) studies revealed that PBTTT thin films are composed of quasi-domains within which the polymer chains are similarly oriented^29^. We assume the major restriction in the charge transport is the quasi-domain boundaries, which can be considered as transport barriers in our model. Adjacent polymer chains can be connected in very complicated ways via the low-angled chain bridging, π-π stacking and side-chain linking. Considering the lack of chain bridging or π-π stacking in the quasi-domain boundaries suppresses the charge transport^29^, the characteristic length for the boundaries is likely to be the interchain spacing along the side-chain direction. For PBTTT solids, the distance between polymer chains in the side-chain direction is determined to be approximately 2.1 nm from X-ray diffraction experiments^30^,^31^. This distance does not exceed 2.3 nm even when the polymers are in a liquid-crystalline phase with melted side chains. Therefore, we assumed *d*_1_ to be 2.1 nm and checked if other variables obtained from the equations are reasonable. All the calculated variables in the multi-barrier model are summarized in Table 1. It is worth mentioning that the concept of multiple identical barriers is not completely new but rather resembles the multi-junction model for microcrystalline silicon although fluctuation-induced tunneling was mainly studied^32^.

To test the generality of this model, we have also fabricated samples using different post-annealing conditions. Two recrystallization rates were adopted to control the microstructure of the PBTTT thin film. The slow-cooled samples have terrace structures hundreds of micrometers in size, whereas the quenched samples have significantly reduced terrace size owing to the insufficient recrystallization time for interdigitation^30^. The power spectrum analysis for atomic force microscopy (AFM) and TEM shows that the characteristic length scales extracted from the two microscopies are closely related to each other and that the quasi-domain size of PBTTT films is also predictable from its terrace dimension in AFM images^29^. The calculated quasi-domain sizes of hundreds of nanometers for slow-cooled samples agree with the previous dark-field TEM studies. The quasi-domain sizes for the quenched films are much smaller than that for the slow-cooled films, which is consistent with the microstructure studies using AFM^30^. Note that the calculated quasi-domain size seems to be nearly independent of the room-temperature mobility of PBTTT thin films and rather closely related to the microstructure of PBTTT thin films.

**Figure 5:**
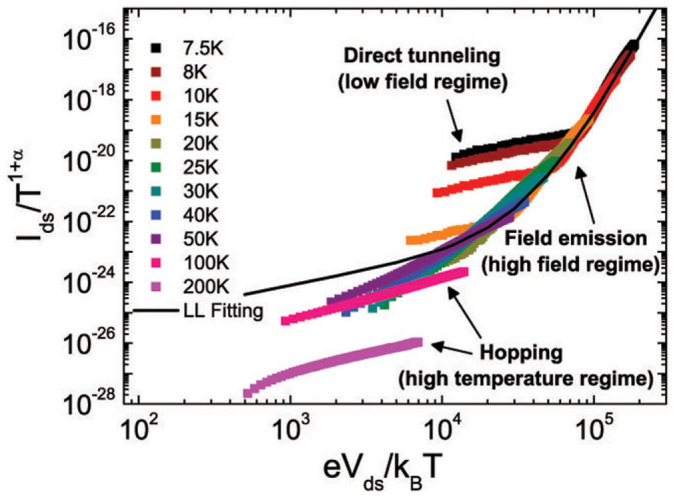
I–V characteristics plotted as *I*_ds_/*T*^1+*α*^ versus *eV*_ds_/*k*_B_*T* for *V*_g_ = −120 V at various temperatures. The black solid line is the best fit to equation (7).

We have also tried to fit the low-temperature I–V characteristics to the LL scaling curve to clarify if the criterion for the data to be well fitted on the scaling curve is the same as that of field emission. LL theory predicts various I–V characteristics can be plotted onto a single universal scaling curve when scaled axes (*I*_ds_/*T*^1+*α*^ versus *eV*_ds_/*k*_B_*T*) are adopted as shown in Figure 5. The scaling curve is given by the equation^3^,^9^:
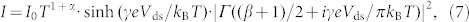
where *α* and *β* are derived from the power-law relations of LL, *σ* ~ *T^α^* at low *V*_ds_ and *I* ~ *V^β^* at low temperatures, respectively, which also satisfy the relation, *α* + 1 = *β*, *γ* is a temperature- and voltage-independent parameter related to the amount of disorder along the 1d path, and *Γ*(*x*) is the gamma function. To construct the best fit to the scaling curve, *α* and *β* were directly obtained from the power-law relations and *I*_0_ and *γ* were used as fitting parameters. The solid fitting line in Figure 5 corresponds to *α* = 8.39, *β* = 9.01, *I*_0_ = 7.5 × 10^−27^ and *γ* = 1/5500. Although the high-*V*_ds_ data are relatively easily collapsed into a single curve, the low-*V*_ds_ tails tend to deviate from the scaling curve. Compared with the Fowler–Nordheim plot, the deviation of these low-*V*_ds_ tails corresponds to the crossover to the direct tunneling regime. In addition, at high temperatures where thermally assisted hopping is dominant, the I–V characteristics break off from the scaling curve. In short, the I–V characteristics can be collapsed into a single curve only if the field emission is the dominant transport mechanism. This suggests that the universal scaling for the transport in PBTTT could originate from the intrachain transport assisted by field emission through multiple barriers along the quasi-1d path. Besides, the exclusive nature of PBTTT is not a requirement for the field-emission phenomenon, as it can appear in other semicrystalline organic thin films with definable domains.

## Discussion

As shown in Figure 4c, we assumed that the quasi-1d transport path between the source and drain electrodes. This simple quasi-1d approach with multiple barriers can be justified by the microstructure of PBTTT thin films. Dark-field TEM and subsequent image analysis also revealed that the orientations of adjacent quasi-domains are mostly found to be low-angled^29^. Within this framework, one can suppose the most probable charge transport to be along the quasi-1d path composed of quasi-domains connected in series with low angles^7^. For high electric fields, the charge-transport dimension is more restricted, promoting quasi-1d transport more effectively^15^. When sufficient carrier density and electric field are available, the probability of tunneling through the domain boundaries can be significantly enhanced. In this regime, the intrachain transport becomes important, which can be accompanied by the weak temperature dependence of conductivity and nonlinearity at low temperatures.

We find the quasi-domain sizes in Table 1 are relatively consistent with the previous microstructure studies^29^,^33^, whereas the obtained values of *ϕ*_0_ seem much greater than the widely known activation energy of tens of eV for PBTTT thin films^8^,^30^. In fact, we believe that *ϕ*_0_ is not identical to the conventional activation energy used within the framework of trap-mediated transport. This value can be rather ascribed to the effective potential energy to overcome a quasi-domain boundary. The difference arises because we assumed quasi-domains are nearly trap-free and the transport is mainly obstructed by the boundaries in the field emission regime.

To clarify the meaning of the parameters, it is significant to remind of the assumptions implied in our model. First, charge-carrier mobility in a quasi-domain is assumed to be much higher than that in a domain boundary, and thus, voltage drop in the quasi-domain was neglected. Recent TEM studies revealed that these large domains do not directly correspond to single crystallites but consist of small crystalline grain flakes (order of 10 nm)^8^,^29^. Because the size of a grain flake is smaller than the length of polymer chains, there should be chain bridging between the grains, which facilitates the quasi-domain formation. Therefore, the barrier between the grain flakes is expected to be small compared to that between the quasi-domains. These small barriers would become more negligible with *V*_g_-induced high carrier densities. Furthermore, considering that the similar orientation of polymer chains in a quasi-domain facilitates intrachain transport and the effective mass for the chain direction (~0.1 *m_e_*) is orders of magnitude lower than that for the π-π stacking direction (~1.6 *m_e_*) or side-chain direction, the voltage drop in the quasi-domain along the transport path will be minor. In addition, no significant voltage drop was assumed near the electrodes due to the contact resistance. We confirmed that the contact resistance obtained in our top contact geometry is small compared to the channel resistance and that the ratio of the contact and channel resistances is not substantially dependent on temperature^34^,^35^. In short, we assumed that the major voltage drop would occur across the quasi-domain boundaries and the contribution from the other voltage drop sources is negligible. Accordingly, the voltage drop across the barriers, which is equivalent to the single barrier height, could be inevitably overestimated in our simple model.

In summary, low-temperature transport in PBTTT thin films was studied. The Fowler–Nordheim plot for the I–V characteristics reveals the crossover from thermally assisted hopping to tunneling with decreasing temperature and the field-induced transition from direct tunneling to field emission at low temperatures. We found a simple model for field emission with multiple barriers can give the average quasi-domain size and barrier height. Because both parameters are calculated for the zero-temperature quasi-domains in which charges can move relatively freely, the quasi-domain size and barrier height might be related to the charge-localization length and charge-localization potential, respectively. Therefore, this analysis could provide a powerful tool to thoroughly understand the transport in conjugated polymers. Application of this analysis method to a wide range of semicrystalline organic materials is necessary.

## Methods

Commercially available PBTTT-C_14_ (Merck Chemical Co.) was used as purchased. The fabrication details have been described in our previous work^30^. PBTTT solution of 0.5 wt% concentration in chlorobenzene was spin cast at 3000 rpm onto a heavily n-doped Si wafer covered with a thermally grown 200-nm-thick SiO_2_ layer. Samples were placed on a metal plate and annealed at 150°C, which is above the liquid-crystalline phase-transition temperature, for 10 min. After annealing, a group of samples was quenched directly on a metal plate with large thermal mass while another group of samples was slowly cooled with cooling rate of 1°C/min. Cooling rate was controlled by a programmable digital hot plate (EchoTherm HS40, Torrey Pines Scientific). FET devices were fabricated with the conventional top-contact geometry. The heavily n-doped Si substrate functioned as a gate electrode. Source and drain electrodes were deposited onto the spin-coated polymer layer by thermal evaporation of Au to a 70-nm-thick layer using a patterned shadow mask. The channel length (L) and width (W) were 20–100 and 2000 μm, respectively.

All electrical measurements were performed with the Keithley 2636 Dual-Channel System Source Meter. We carefully organized the measurement system with a leakage current less than 10^−10^ A for each temperature range. For temperature-dependent transport measurements, samples were mounted on the cold head of a GM refrigerator (130 GM, Leybold Cryogenics), where a Si thermometer (LakeShore Cryotronics) was attached. Each measurement was performed after thermal equilibrium was attained. After the low-temperature measurement, the samples were tested at room temperature again, and they were found to exhibit typical transistor characteristics with slightly decreased mobility. Although 200-nm-thick SiO_2_ and channel lengths of tens of micrometers can allow for a fairly high voltage difference up to 150 V at low temperatures, we chose 120 V as the maximum voltage for sustainability. However, some samples became shorted even for voltages less than 120 V at relatively high temperatures of approximately 200 K.

## Author Contributions

E.K. designed the project and experiments. E.S.H.K. carried out the experimental work and data analysis. E.S.H.K. and E.K. wrote the manuscript.

## Figures and Tables

**Table 1:** Calculated variables in the multi-barrier field-emission model

Recrystallization rate	Channel length *L* (μm)	RT mobility (cm^2^ V^−1^ s^−1^)	Single barrier height *ϕ*_0_ (eV)	Domain size *d*_1_ + *d*_2_ (nm)	Number of barriers (#)
slow	20	6.51 × 10^−2^	0.617	418	48
slow	30	1.67 × 10^−1^	0.524	393	76
slow	40	1.10 × 10^−1^	0.337	197	203
quench	30	5.06 × 10^−2^	0.425	164	183
quench	40	4.72 × 10^−2^	0.209	85	471
